# Avidin related protein 2 shows unique structural and functional features among the avidin protein family

**DOI:** 10.1186/1472-6750-5-28

**Published:** 2005-10-07

**Authors:** Vesa P Hytönen, Juha AE Määttä, Heidi Kidron, Katrin K Halling, Jarno Hörhä, Tuomas Kulomaa, Thomas KM Nyholm, Mark S Johnson, Tiina A Salminen, Markku S Kulomaa, Tomi T Airenne

**Affiliations:** 1 NanoScience Center, Department of Biological and Environmental Science, P.O. Box 35 (YAB), FI-40014 University of Jyväskylä, Finland; 2 Department of Biochemistry and Pharmacy, Åbo Akademi University, Tykistökatu 6 A, FI-20520, Turku, Finland

## Abstract

**Background:**

The chicken avidin gene family consists of avidin and several avidin related genes (*AVR*s). Of these gene products, avidin is the best characterized and is known for its extremely high affinity for D-biotin, a property that is utilized in numerous modern life science applications. Recently, the *AVR *genes have been expressed as recombinant proteins, which have shown different biotin-binding properties as compared to avidin.

**Results:**

In the present study, we have employed multiple biochemical methods to better understand the structure-function relationship of AVR proteins focusing on AVR2. Firstly, we have solved the high-resolution crystal structure of AVR2 in complex with a bound ligand, D-biotin. The AVR2 structure reveals an overall fold similar to the previously determined structures of avidin and AVR4. Major differences are seen, especially at the 1–3 subunit interface, which is stabilized mainly by polar interactions in the case of AVR2 but by hydrophobic interactions in the case of AVR4 and avidin, and in the vicinity of the biotin binding pocket. Secondly, mutagenesis, competitive dissociation analysis and differential scanning calorimetry were used to compare and study the biotin-binding properties as well as the thermal stability of AVRs and avidin. These analyses pinpointed the importance of residue 109 for biotin binding and stability of AVRs. The I109K mutation increased the biotin-binding affinity of AVR2, whereas the K109I mutation decreased the biotin-binding affinity of AVR4. Furthermore, the thermal stability of AVR2(I109K) increased in comparison to the wild-type protein and the K109I mutation led to a decrease in the thermal stability of AVR4.

**Conclusion:**

Altogether, this study broadens our understanding of the structural features determining the ligand-binding affinities and stability as well as the molecular evolution within the protein family. This novel information can be applied to further develop and improve the tools already widely used in avidin-biotin technology.

## Background

Avidin from eukaryotic chicken together with streptavidin from prokaryotic *Streptomyces avidinii *share an unique property not seen in any other known proteins, an extremely high affinity (K_d _≈ 10^-15 ^M) to its natural ligand, the water-soluble vitamin D-biotin [1,2]. High affinity and stability of the free and complex forms of (strept)avidin and biotin [1], the easy attachment of biotin to various target molecules [3] and the non-obtrusive chemical nature of biotin are currently exploited in numerous (strept)avidin-biotin based life science applications [4].

Avidin is postulated to exist throughout the oviparous vertebrates [5-7] and has long been known to be the operational biotin-harvester in chicken egg-white, comprising about 0.05% of the total protein [1]. Recently, avidin related genes (*AVR*s) highly similar to avidin (91–95% identity at the nucleotide level) have also been found in the chicken genome [8-10], suggesting that in addition to avidin AVRs may also play a role in biotin-harvesting. *AVR*s seem to have functional promoter regions [11] and are located in close vicinity of the avidin gene on the chicken male-sex chromosome Z [10,12]. Interestingly, the total number of *AVR*s is likely to vary between different individuals and even between different cells of the individual chicken [13]. The function of the *AVR *gene products is unknown, however, since the proteins encoded by them have not yet been isolated from chicken although mRNAs of *AVR1, AVR2 *and *AVR3 *are found during inflammation [11].

Since the avidin gene of chicken is the only cloned avidin gene within the vertebrates [14], structure-function and protein engineering studies have long been concentrated on it [15-24]. The three-dimensional structure of chicken avidin has already been determined, too [25,26]. The bacterial homolog of avidin, streptavidin, is also well characterized: the gene encoding steptavidin has been cloned [27], its structure solved [28] and several studies on the biochemical properties and protein engineering of streptavidin have been reported [17,29-34].

In order to characterize the proteins encoded by the *AVRs*, avidin related proteins (AVRs) have recently been produced in insect cells using a baculovirus expression system and have been demonstrated to be functional biotin-binding proteins like chicken avidin [35]. AVRs do, however, show unique features when compared to avidin. The AVRs differ from avidin, with respect to glycosylation and charge properties, and all AVRs except AVR2 contain an uneven number of cysteine residues in their sequence, which can form inter-subunit disulphide bridges in addition to the intra-subunit disulphide bridges also seen in avidin [35]. Interestingly, the biotin-binding affinities of AVRs have been reported to vary over a wide range of values, AVR4 being almost as efficient a biotin binder as avidin [35,36] and AVR2 showing the lowest affinity for biotin [35]. AVRs, like avidin, have been found to be very stable proteins, too; AVR4 has clearly higher thermal stability than avidin [35,36]. Recently, we have been able to produce avidin and some AVRs in *Escherichia coli*, too [37]. The 3D-structure of AVR4 has been recently determined [38], and we have been able to produce chimeric forms of avidin and AVR4, which retained their high biotin affinity and showed improved thermal stability [39].

In this study, we have determined the high-resolution X-ray crystal structure of AVR2 in complex with the natural ligand, D-biotin. By using site-directed mutagenesis and recombinant expression techniques combined with structural studies, we have been able to characterize some of the structural factors responsible for the varying biochemical properties of the members of the chicken avidin protein family. The results may be utilized in avidin protein engineering aiming to fine-tune the ligand binding properties and thermal stability of AVRs and their chimeric forms in experiments needed to expand the tools available in the area of avidin-biotin technology. This study provides insight into the molecular evolution within the avidin family, too.

## Results

### Production and mutagenesis of proteins

**Figure 1:**
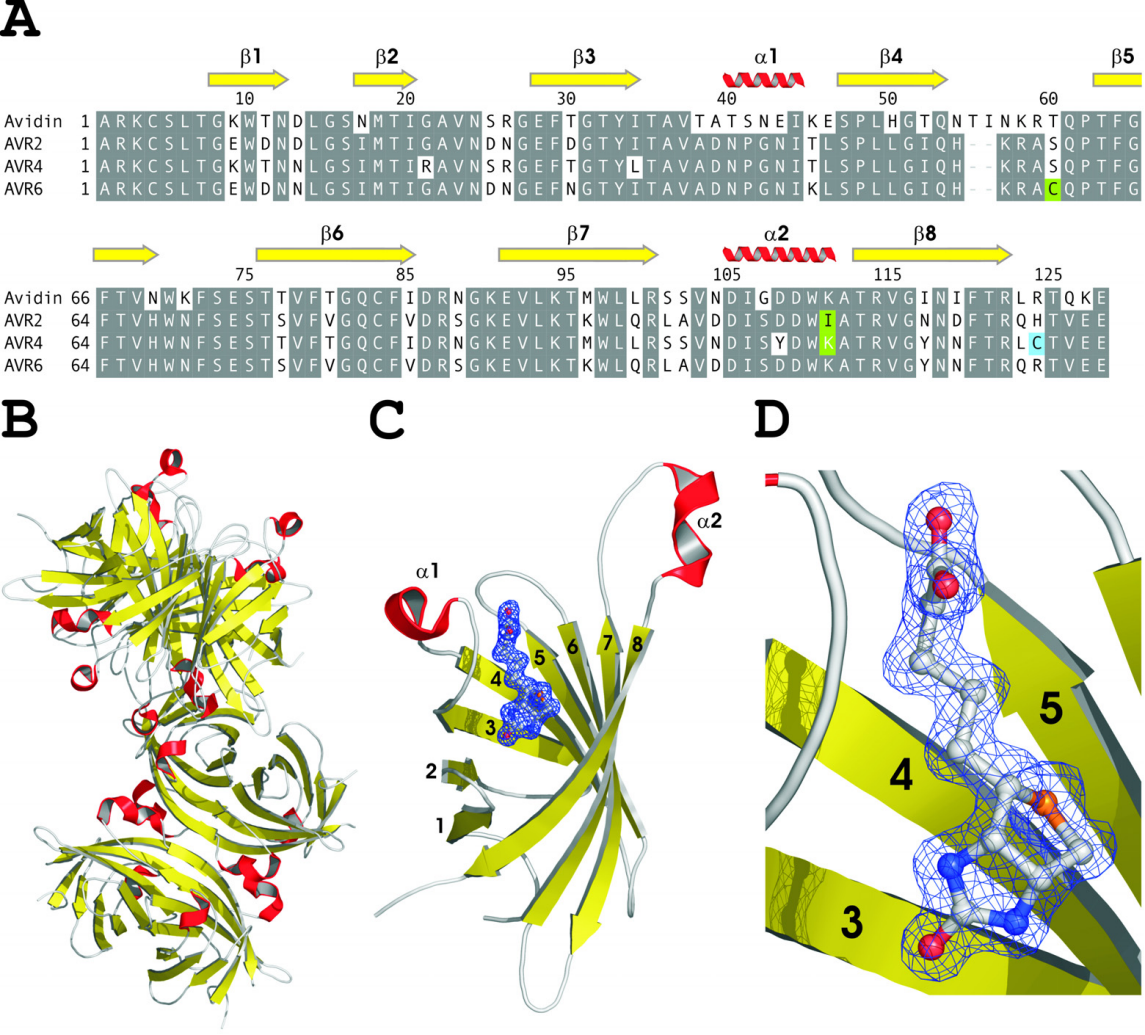
**The X-ray structure of AVR2. **(A) Multiple sequence alignment of the avidin family. The secondary structure elements are numbered according to the AVR2 structure. The residues mutated in this study (AVR2, Ile-109; AVR4, Lys-109; AVR6, Cys-58) are coloured green. Residue Cys-122 in AVR4, which was mutated to serine in a previous study [36], is indicated in blue. (B) Two tetramers found in the asymmetric unit of the AVR2-b crystal. (C) Monomer A of AVR2-b. The weighted difference *F*_o _– *F*_c _electron density map (blue), calculated in the absence of biotin, is drawn with a 1.5 Å radius around the atoms of D-biotin of the final structure of AVR2-b. Contours are shown at 3.0σ. The secondary structure elements of AVR2-b are numbered. (D) A close-up view of (C) focused on biotin.

Several different AVRs, AVR2, AVR2(I109K), AVR4(K109I), AVR6, and a novel avidin mutant K111I, were efficiently expressed either in insect cells or using a bacterial expression system as summarised in Table 1 [37,40]. Since wild type AVR6 was found to form oligomers in solution *via *disulphide bridges (data not shown), Cys-58 of AVR6 was mutated to Ser based on the alignment of AVR6 with AVR2 and AVR4 (Figure 1). The resulting protein AVR6(C58S) (hereafter referred to AVR6), as well as all of the other studied proteins, were found to form avidin-like tetramers according to analysis by size exclusion chromatography (Table 2) and confirmed to be pure and homogenous using SDS-PAGE. The elution time of AVR2 was slightly different from that of avidin and of the other studied AVRs, which can partially be explained by the different charge properties of these proteins: AVR2 has a theoretical isoelectric point of 4.7 compared to 9.6 for AVR4, 6.9 for AVR6 and 9.7 for avidin. The effect of glycosylation is also clearly seen by the varying elution times in gel filtration analysis (Table 2) for AVR2-b produced in bacteria (no glycosylation) and from insect cells (two potential glycosylation sites; both utilized to some extent [35]).

Purification of AVR2 and AVR6 on the 2-iminobiotin column was inefficient and therefore biotin affinity chromatography was used to isolate these proteins. In order to confirm the quality of the protein and correct cleavage of the bacterial signal peptide used in the production of AVRs in *E. coli*, the molecular weight of AVR2-b was determined using ESI-MS. The M_r _(14310.0 ± 0.3 Da) determined from the experimental data using four charge states correlates well with the expected M_r _of 14307.8 Da. The final product from *E. coli *expression carries three additional residues (QTV) at the N-terminus as previously reported for avidin produced using the same method [37].

In a previous study, the K109I mutation of AVR2 was hypothesised to be at least partially responsible for the lower biotin-binding affinity of AVR2 when compared to other AVRs and avidin [35]. In order to validate this suggestion, we subjected AVRs to mutagenesis. The mutation I109K was introduced into AVR2 to increase its biotin-binding affinity. Likewise, the mutation Lys→Ile was introduced into both AVR4 and avidin in order to cross-validate the hypothesis and lower the biotin-binding affinity of AVR4 and avidin. Gel filtration analysis showed that all of the mutated proteins corresponded to tetramers (Table 2).

### X-ray structure of AVR2

The X-ray structure of AVR2-b in complex with D-biotin was determined at 1.4 Å resolution. The structure determination statistics are summarized in Table 3. As expected based on the sequence alignment (Figure 1), AVR2 has an overall three-dimensional structure similar to those of avidin [PDB:1AVD] [26] and AVR4 [PDB: 1Y52] [38]. Each monomer in the homotetrameric protein has a β-barrel fold of eight β-strands with one end of the barrel adapted to bind D-biotin (Figure 1C).

Although AVR2 shares many structural features with avidin and AVR4, there are clear differences in their functional properties (Table 2, Figure 4). The most distinctive structural differences are found around the terminal carboxylate group of D-biotin. In avidin, atom O10B of the terminal carboxylate group of D-biotin is hydrogen bonded to the backbone nitrogen atoms of Ala-39 and Thr-40 of the L3,4 loop [26], whereas in AVR4 there is only one polar contact between the O10B atom and the backbone nitrogen atom of Asp-39 of the L3,4 loop [38]. In AVR2, the O10B atom of D-biotin forms an additional hydrogen bond to the NE2 atom of Gln-97. Moreover, in avidin, the O10A atom of the carboxylate group of biotin is hydrogen bonded to the OG atoms of Ser-73 and Ser-75, while in AVR2 and AVR4 there is only one hydrogen bond to the OG atom of Ser-71 (equivalent to Ser-73 in avidin).

**Figure 2:**
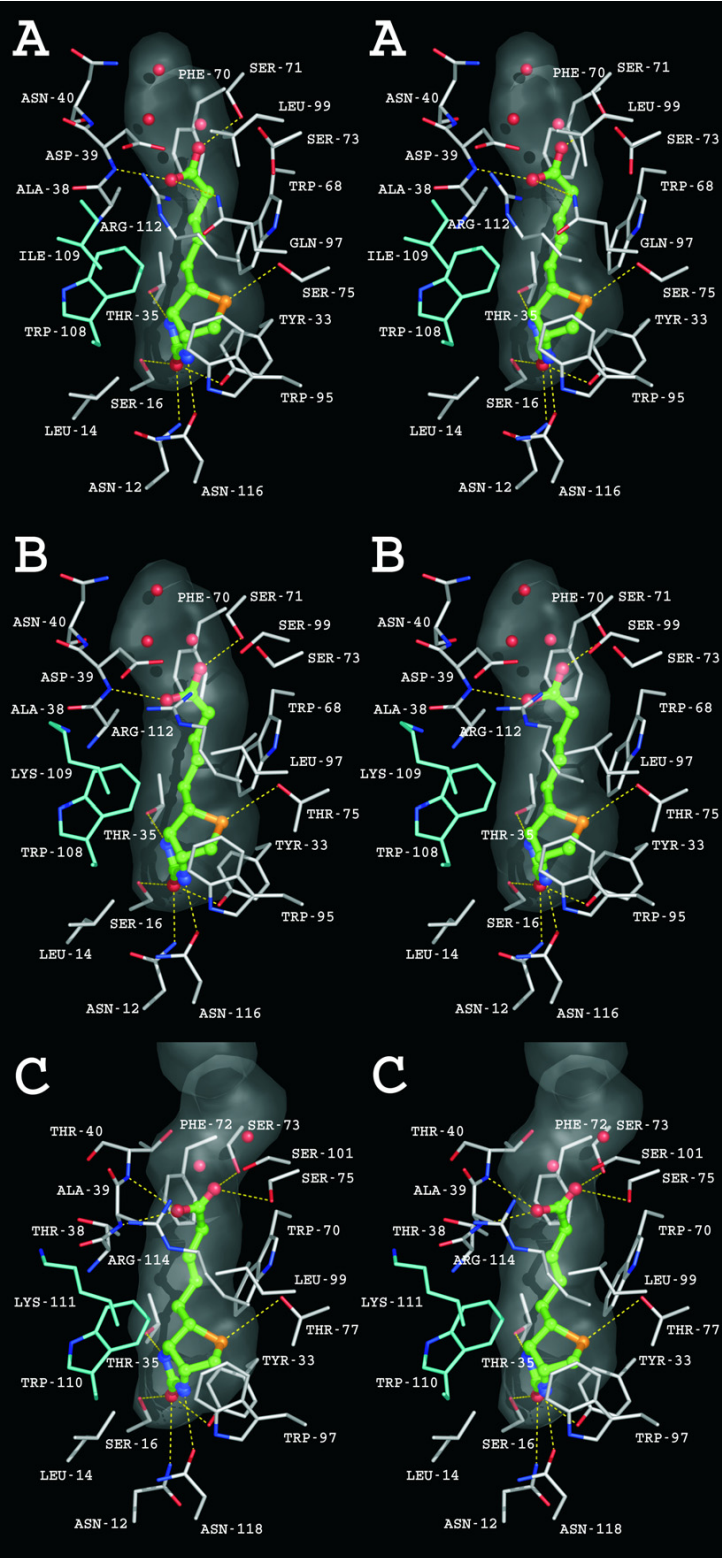
**Biotin-binding sites of avidin, AVR2 and AVR4. **Stereo images of the biotin-binding sites of (A) AVR2-b, (B) AVR4 [PDB:1Y52] [38] and (C) avidin [PDB:1AVD] [26] are shown. The cavities (transparent) around the bound biotin molecules of subunit A are shown. The water molecules within the cavities are shown as red spheres. Hydrogen bonds between the atoms of D-biotin and the surrounding proteins are shown with dashed lines. Carbon atoms of residues from subunit 1 are coloured white; those from a neighbouring subunit 2 are indicated in cyan: residues 108 and 109 (110 and 111 in avidin).

A few differences are also found around the central aliphatic segment and the bicyclic ring system of D-biotin. Leucine in avidin (Leu-99) and AVR4 (Leu-97) is replaced by glutamine in AVR2 (Gln-97), and threonine in avidin (Thr-77) and AVR4 (Thr-75) is replaced by Ser-75 in AVR2. The side-chain atoms of Gln-97 of AVR2 not only interact with the valeryl segment of D-biotin, as in the case of the corresponding Leu in avidin and AVR4, but also with the carboxylate group of D-biotin (see above). The corresponding residue in AVR4 and avidin is leucine, but glutamine is also found aligned at the equivalent position in other AVRs. The presence of the polar head group of Gln-97 in AVR2 also affects the type and conformation of the neighbouring residues, such as Ser-73, Leu-99 and Arg-112 as well as Ile-109 from another subunit. These residues line the entrance of the biotin-binding pocket: i) Ser-73 of AVR2 has two alternative rotamers unlike in the X-ray structure of avidin and AVR4, ii) Leu-99 of AVR2 is replaced by Ser-101 in avidin (Ser-99 in AVR4), iii) Arg-112 of AVR2 and AVR4 form a salt bridge with Asp-39 from the L3,4 loop, but Arg-112 has a different conformation in AVR2 than in AVR4 (in avidin the corresponding ARG-114 does not form a salt bridge with any of the residues from the L3,4 loop) and iv) Ile-109 of AVR2 is substituted by Lys-111 in avidin and by Lys-109 in AVR4. In addition, Ile-109 of AVR2 is located next to Trp-108, a residue known to be important for biotin binding [17,30], but Ile-109 does not appear to alter the conformation of this residue significantly in comparison to avidin and AVR4. All of these differences are located at the open end of the biotin-binding pocket and are very likely responsible for the varying biotin binding properties of avidin, AVR2 and AVR4. Moreover, although D-biotin adopts a very similar conformation in avidin, AVR2 and AVR4, the biotin-binding network is not identical (Figure 2).

**Figure 3:**
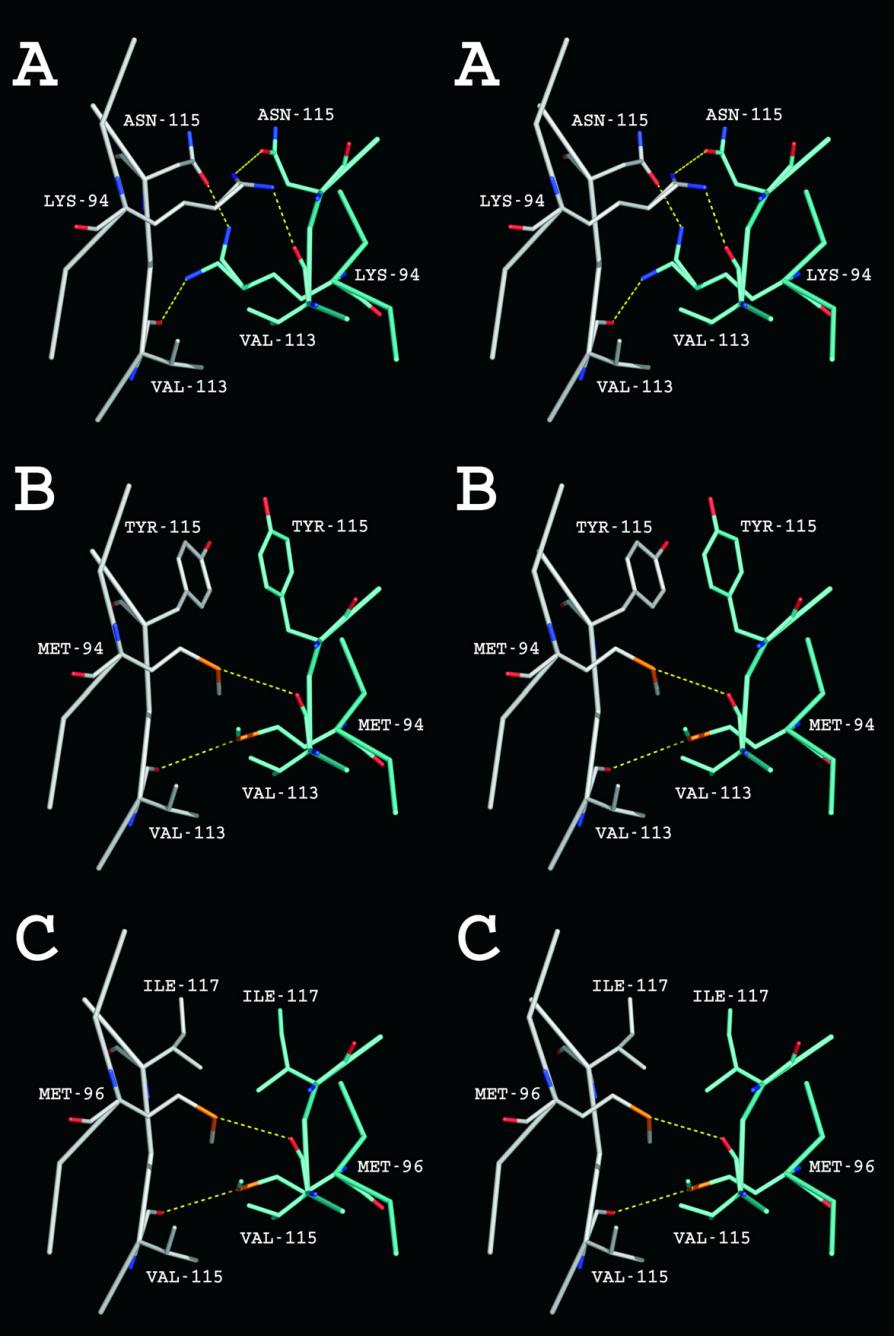
**The 1–3 subunit interface of AVR2, AVR4 and avidin. **Stereo images of the 1–3 subunit interface of (A) AVR2-b, (B) AVR4 [PDB:1Y52] [38], and of (C) avidin expressed in *E. coli *[PDB:1VYO] (Airenne, Hytönen *et al. *unpublished) are shown. Lys-94 of AVR2 exists in two alternate conformations, which can form a hydrogen bond with the side-chain oxygen atom of Asn-115 or with the main-chain oxygen atom of Val-113, both from an adjacent subunit. Putative hydrogen bonds are shown by dashed lines. The carbon atoms of subunits 1 and 3 are coloured white and cyan, respectively.

The 1–3 subunit interface (numbering according to Ref. [25]) is also markedly different in amino acid composition in AVR2 in comparison with avidin and AVR4. In avidin, the subunit contacts are established between the hydrophobic amino acid residues Met-96, Val-115 and Ile-117 [25,26], which are in contact with the same set of residues from a neighbouring subunit. In AVR2, only valine (Val-113 in the AVRs) is conserved, while Met-96 and Ile-117 of avidin are respectively substituted by the hydrophilic residues Lys-94 and Asn-115 in AVR2 (Figure 1A). In the crystal structure of AVR2, the side chain of Lys-94 can exist in two alternate rotamers, which are hydrogen bonded either to the side-chain oxygen atom of Asn-115 or to the main-chain oxygen of Val-113, both from a neighbouring subunit (Figure 3). Asn-115 is also in contact with Asn-115 from a neighbouring subunit. In AVR4, Ile-117 of avidin is substituted by Tyr-115, the latter interacting strongly with Tyr-115 of an adjacent subunit and through a hydrogen bond to Lys-92 [38].

### Comparative analysis of avidin family proteins

Previously, it has been found that AVR6 forms intermonomeric disulphide bridges [35]. Gel filtration analysis of AVR6 revealed that these disulphide bonds are formed between tetramers, thus causing further oligomerization of the protein (not shown). Consequently, we introduced the C58S mutation into AVR6, which successfully blocked oligomerisation, and used this mutated protein form in the comparative analyses in the present study.

The overall charge of AVR2 (pI ≈ 5) is very different when compared to that of avidin and AVR4 (pI ≈ 10). The number of ionic bonds in avidin is seven per subunit, whereas three salt bridges are seen in AVR4 [38]. In the AVR2-biotin complex, four intra-subunit salt bridges are detected: Asp-39-Arg-112, Glu-89-Arg-120, Lys-92-Asp-117 and Arg-98-Asp-107.

### Biotin dissociation analysis

**Figure 4:**
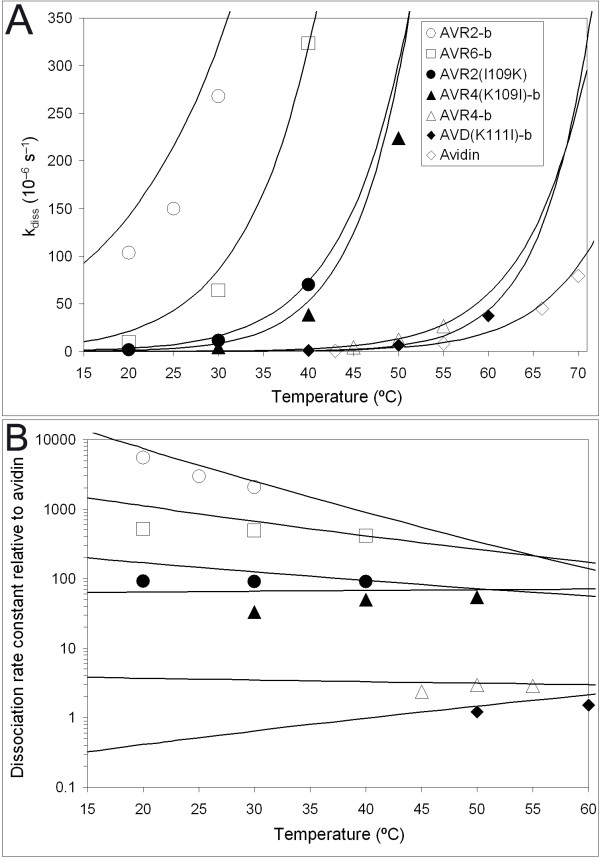
**Biotin dissociation analysis. **(A) Temperature-dependence of biotin dissociation rates measured by the [^3^H]biotin dissociation assay. Radiobiotin dissociation rate constants measured for the proteins are plotted as a function of temperature. The models for dissociation rate constants obtained by global fit analysis [41] are shown by lines and the determined individual dissociation rates by symbols. The dissociation rate constants determined previously for avidin and AVR4-b are also shown [39]. (B) Relative dissociation rate constants of the AVRs and avidin mutant K111I compared to that of avidin. The individual dissociation rates as well as global fit analysis models of the dissociation rate are divided by the dissociation rate constants of avidin obtained from global fit analysis. The data are plotted using a logarithmic y-axis.

Of the proteins studied, the fastest [^3^H]biotin dissociation rate was found with AVR2, while the slowest rate was measured for avidin (Figure 4). Ile-109 is found close to the biotin-binding site in AVR2, whereas all other proteins in avidin family [35] have lysine at the equivalent position. In order to test the effect of Ile-109 on the biotin dissociation rate, the AVR2(I109K) mutant was produced. The resulting mutant had a significantly slower dissociation rate than the wild-type protein. AVR6, in turn, showed a dissociation rate constant in between the values observed for the two AVR2 forms. The dissociation rate constant for AVR4, measured in a previous study [39], was somewhat higher when compared to that of avidin. When Lys-109 of AVR4 was mutated to isoleucine according to the sequence of AVR2, the rate of dissociation increased as expected, but biotin binding of the resulting protein was still stronger than for the mutated AVR2 form. Similarly, the analogous mutation K111I in avidin increased the biotin dissociation rate compared to the wild-type protein. Hence, the analyzed proteins can be sorted according to their biotin-binding affinities (as the biotin dissociation rate decreases, biotin binding strengthens): AVR2 < AVR6 < AVR2(I109K) < AVR4(K109I) < AVR4 < AVD(K111I) < avidin (Figure 4).

The biotin dissociation data, measured at various temperatures, were analysed using the global fit method described elsewhere [41]. The resulting dissociation rate temperature-dependency model was compared to the one previously measured for avidin [39]. The analysis revealed a different temperature-dependency for AVR2 in comparison to avidin (Figure 4B). The mutation I109K caused a shift in the temperature-dependency of AVR2-biotin dissociation, resulting in a model resembling that determined for AVR6. AVR4 has a similar temperature-dependency of the biotin dissociation rate as avidin, and the K109I mutation did not significantly change the temperature-dependency although it clearly increased the biotin dissociation rate constant (Figure 4B). The equivalent mutation K111I in avidin resulted in a nearly two-fold increase in the biotin dissociation rate over a temperature range of 40–60°C (Figure 4B).

### Differential scanning calorimetry

The thermostability of avidin, AVR2, AVR4 and AVR6 were measured using DSC analysis (Table 2). In this analysis, AVR2 showed higher thermostability than avidin. The measured T_m _(91.3°C) was between the values measured previously for avidin (83.5°C) and AVR4 (106.4°C) [36]. As expected, the thermal stability of AVR2 increased in the presence of biotin (T_m _= 112.5°C), similarly as reported for avidin, AVR4 and streptavidin [22,36,42]. The I109K mutation significantly stabilised AVR2, resulting in a 6.3°C increase in T_m _as compared to the wild-type protein. The reverse mutation, K109I in AVR4 and K111I in avidin, led to destabilisation of the proteins, resulting in a 1.9°C and 7.0°C decrease in the T_m_, respectively. Interestingly, AVR6 showed slightly higher thermal stability (T_m _= 87.7°C) than avidin (T_m _= 83.5°C) in the absence of biotin, while in its presence the T_m _of AVR6 (114.0°C) was raised significantly but remained lower than that measured for the avidin-biotin complex (T_m _= 117.0°C).

## Discussion

In the present study, we have used targeted mutagenesis and X-ray crystallography combined with the comparative analysis of thermal stability and ligand-binding kinetics to dissect the functional properties of the chicken avidin protein family. The high-resolution structure of AVR2, a close relative of avidin, provides new insights into the biotin-binding mechanism of the avidins and serves as a new source of knowledge for protein engineering studies, too.

In order to understand the observed differences in the biotin-binding affinities and thermal stabilities within the avidin protein family, the crystal structures of avidin [26], AVR2, and AVR4 [38] were compared, all in complex with D-biotin. Overall, these proteins share high structural similarity and their ligand-binding sites within the eight-strand β-barrel resemble each other. The most distinctive structural differences are found around the terminal carboxylate group and central valeryl segment of D-biotin. In the AVR2 structure, D-biotin is in contact with the L3,4 loop as in the case of avidin [26] and AVR4 [38], but also in contact with the side-chain atom of Gln-97 unlike the avidin or AVR4 complexes where leucine is found at the equivalent position. Glutamine is conserved in all of the AVRs except for AVR4 [35], resides 3.3 Å away from D-biotin in the AVR2 structure, and a hydrogen bond may form between Gln-97 and biotin even though the angle is not optimal. In addition to D-biotin, Gln-97 seems to form a hydrogen bond with Ser-73, which exists in two alternative conformations. The presence of Gln-97 in AVR2 probably affects the conformation of nearby Arg-112, which is slightly displaced with respect to the corresponding residue seen in AVR4 and avidin. Then again, the conformation of Arg-112 may be altered due to interactions with the AVR2-specific Ile-109, too. Ile-109 of AVR2 respectively corresponds to Lys-109 and Lys-111 in avidin and AVR4. In AVR2, Ile-109 resides close to Trp-108, which is known to be important for biotin binding [17,30], but the conformation of Trp-108 does not seem to be significantly affected by Ile-109. Yet another sequence difference, whereby Thr-77 of avidin and the corresponding threonine of AVR4 (residue 75 in the AVRs) is substituted to Ser in AVR2 does not appear to disrupt hydrogen bonding to the sulfur atom of D-biotin. However, this substitution enlarges the binding cavity around bound biotin (Figure 2) and hence contributes to the lower affinity of AVR2 for biotin. The biotin-binding network is not identical in avidin, AVR2 and AVR4 despite the similar conformation that D-biotin adopts in all of these structures. In general, the polar contacts with D-biotin seem to be much more variable than the hydrophobic ones, which are highly conserved, indicating their important role in the biotin-binding process. In line with this, the importance of hydrophobic residues for biotin-binding of streptavidin has been demonstrated experimentally [30]. Moreover, the interactions of avidin, AVR2 and AVR4 with the carboxylate group of D-biotin are clearly less conserved than the interactions with the central aliphatic valeryl segment and the bicyclic ring system of the tetrahydrothiophenic and ureido rings buried deeper within the biotin binding pocket [26,38,43].

Based on the temperature-dependence of the biotin dissociation rates and relative biotin dissociation rate constants (Figure 4), the order of the biotin binding affinities is as follows: AVR2 < AVR6 < AVR4 < avidin. These results are in line with the previous ligand-binding analyses performed for AVRs using an optical biosensor [35]. Furthermore, the presence of an isoleucine residue at sequence position 109 in AVR2 rather than lysine seems to be the most dominant difference affecting biotin binding in comparison to AVR6. However, this sequence variation does not explain the differences in the biotin-binding properties of AVR2 *versus *AVR4. This was confirmed by analysing the AVR4(K109I) mutant, which showed significantly stronger interactions with biotin when compared to wild-type AVR2. Moreover, the equivalent mutation K111I in avidin affected only slightly the dissociation rate constant of avidin. The temperature-dependency model suggests even slower dissociation rates at low temperatures for the mutant compared to wild-type avidin (Figure 4B). The different effects of the Lys→Ile mutation on avidin *versus *AVR4 may reflect differences at the L3,4 loop of avidin and AVRs. Although the AVR2-biotin dissociation rate was over 5000-fold higher at 20°C than that of avidin and thus showed significantly lower biotin-binding affinity than avidin, the thermal stability of AVR2 in the absence of biotin is higher (T_m _= 91.3°C) than for avidin (T_m _= 83.5°C). Higher thermal stability is notable and may have a functional role.

The biological role of AVRs is unclear; avidin is thought to work as a biotin-harvester in chicken egg-white, thus preventing growth of biotin-dependent organisms [1]. The lower biotin-binding affinity and higher stability of AVR2 raises the question if AVR2 has any biological role similar to avidin. The expression of avidin is induced in chicken during inflammation in various tissues and mRNAs of some AVRs, including AVR2, have been detected during inflammation [11]. This suggests that AVR2 (and the other AVRs) may play a role in inflammatory reactions.

The conformation of the L3,4 loop of AVR2 was found to strongly resemble that of AVR4 [38]. In avidin, this loop is disordered in the absence and ordered in the presence of D-biotin [25]. In contrast, the L3,4 loop of AVR4 was previously found to be in an nearly identical, fixed conformation both in the absence and presence of D-biotin [38]. The latter situation seems to be true for all AVR proteins, since the L3,4 loop *per se*, as well as the neighbouring sequences between the β3 and β5 strands, are highly conserved within the AVR family but quite different from avidin [35]. Recently, this region was transferred to avidin from AVR4 [39]. The resultant chimeric ChiAVD showed better thermal stability (T_m _= 96.5°C) than avidin (T_m _= 83.5°C) [39] and, interestingly, the observed stability of AVR2 (T_m _= 91.3°C) and its mutant AVR2(I109K) (T_m _= 97.6°C) was similar to that of ChiAVD. Hence, in addition to the 1–3 subunit interface, the region between the β3 and β5 strands of AVR2 is likely to affect the stability of the protein (Figure 3). This view is supported by our preliminary results of engineered dual chain avidins suggesting only slightly better stability for the AVR2-type 1–3 interface compared to the 1–3 interface of avidin (Hytönen *et al. *unpublished results).

**Figure 5:**
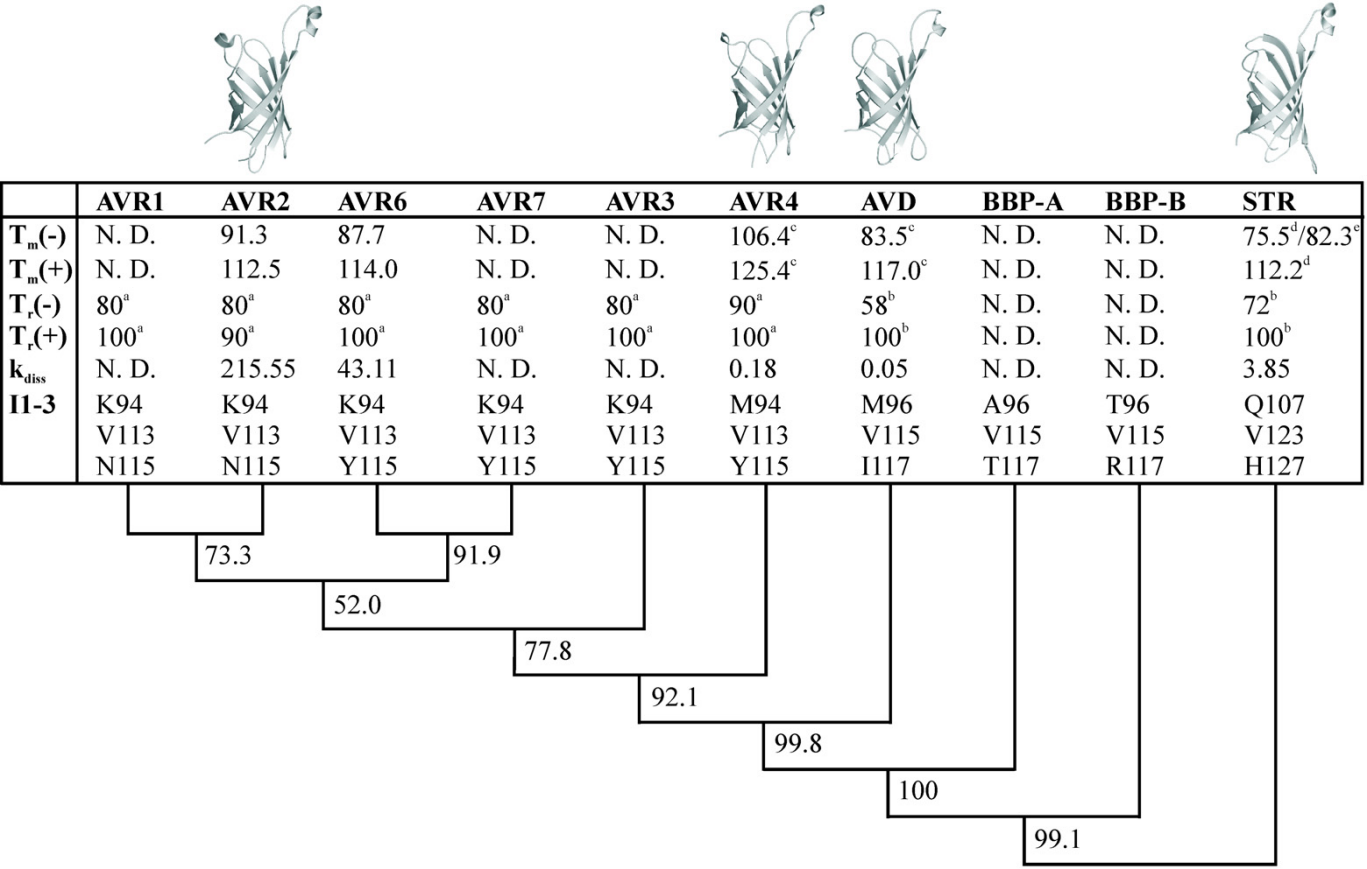
**Summary of the biochemical properties and phylogeny of avidin (AVD) and the AVRs. **The putative chicken biotin-binding proteins BBP-A and BBP-B [12] as well as streptavidin (STR) are included. The confidence levels of the branching of the phylogenetic tree were assessed using the bootstrap method [64]. The tree is unrooted and the branch lengths are not to scale. The values at each node represent the percentage of the 1000 trees where the species above the node are consistently found. T_m_(-), heat-induced unfolding temperature without D-biotin (°C). T_m_(+), heat-induced unfolding temperature in the presence of D-biotin (°C). T_r_(-), transition temperature of oligomeric disassembly without D-biotin determined using SDS-PAGE assay (°C) [70]. T_r_(+), transition temperature of oligomeric disassembly in the presence of D-biotin (°C). k_diss_, biotin dissociation rate constant (× 10^-6 ^s^-1^) obtained from global fitting [41] of the ^3^H-biotin dissociation data. I1–3, three residues important for the 1–3 subunit interface (equivalent to residues 94, 113 and 115 in AVR2). The residues in BBP-A and BBP-B are numbered according to avidin. N. D., not determined. ^a^Hytönen VP, unpublished data. Analysis performed on wt proteins produced in the baculovirus expression system as described in Ref. [35]. ^b^From Ref. [70]. ^c^From Ref. [36]. ^d^From Ref. [71]. ^e^From Ref. [72].

The present study provides novel knowledge of the structural characteristics of AVR proteins. Avidin related proteins are considered as an individual branch in the evolutionary tree of avidins in chicken [44]. Based on the results of the current study (Figure 5), AVR2 and AVR6 seem to be functionally closely related to each other, supporting the previous phylogenetic analysis [44]. It seems that the critical difference, isoleucine at position 109 in AVR2 in comparison to lysine in avidin and the other AVRs, has arisen after the divergence of AVR4 and the rest of AVRs. This sequence difference also explains why AVR2 has the lowest observed biotin-binding affinity among the AVRs. All AVRs have a region between β3 and β5 strands, which is quite different from that in avidin [35]. In comparison with the other AVRs, this region in AVR4 shares a higher level of similarity with avidin. This likely correlates with the high, avidin-like biotin-affinity of AVR4 *versus *the lower biotin-affinity of the other AVRs [39].

Ligand binding to avidin and streptavidin can be considered as an extreme discovery of nature in the sense of affinity and free energy [45]. The structural complementarity between biotin and its binding site in (strept)avidin is almost perfect, which together with the numerous hydrogen bonds that are formed between (strept)avidin and biotin is the basis for the extraordinary tight binding [25,28]. Thus, it is not surprising to find that a small perturbation in this highly perfected system can reverberate as a major change in the biotin binding kinetics. It is known that the high biotin-binding affinity of (strept)avidin is dominated by extremely slow ligand dissociation rates, especially in the case of avidin.

## Conclusion

The high-resolution structure of AVR2 combined with the ligand binding data broadens our understanding of the general principles of ligand-binding processes. Furthermore, the structural information can be employed as a basis to create improved tools for biotechnology. This was demonstrated in a previous study, where chimeric forms of avidin and AVR4 showed improved properties compared to the native proteins [39].

## Methods

### Production and mutagenesis of proteins

Proteins were expressed using the Bac-to-Bac baculovirus expression system in Sf9 insect cells in biotin-free media as previously reported [40]. Bacterial expression in BL-21(AI) (Invitrogen) was also used for protein expression as described in Ref. [36]. The proteins were isolated using affinity chromatography with an 2-iminobiotin or biotin matrix (Affiland S. A., Belgium) as described earlier [17]. Biotin was used as the capture ligand for AVR2, AVR2-b, AVR2(I109K) and AVR6-b, and for these proteins elution was achieved using acetic acid. The recombinant proteins investigated in this article are summarised in Table 1.

### Crystallization and data collection

Minimal Screen 12 [46], a sparse matrix protein crystallization screen [47], was used to search for suitable conditions for crystallization of AVR2-b with the vapor diffusion hanging drop method at 22°C. An orthorhombic crystal with approximate dimensions of 0.15 × 0.1 × 0.1 mm was obtained within three weeks using equal volumes (1 μl) of sample solution containing 0.5 mg/ml protein in 50 mM NaPO_4 _(pH 7.0), 100 mM NaCl and well solution containing 0.1 M Na-citrate (pH 4.6) and 1.5 M NH_4_PO_4_. Before crystallization, the AVR2-b – biotin complex was prepared by adding biotin to the protein solution in a molar ratio of 5:1, respectively, followed by incubation at 4°C for 1.5 hours. For data collection, the AVR2-b crystal was cryoprotected with 20% glycerol (v/v) and 2 M lithium sulfate just prior to flash-freezing in a 100 K nitrogen stream (Oxford Cryostream). Diffraction data were collected from a single crystal at the ESRF beam line ID14-1, Grenoble at 100 K using an ADSC Q4R CCD detector. Data were processed with programs of the XDS program package [48]. Data collection statistics are summarized in Table 3.

### Structure determination

The X-ray structure of AVR2-b was solved using the molecular replacement method and programs from the CCP4i suite [49]. The space group (*P*2_1_2_1_2_1_) of the AVR2-b crystal was ascertained by Amore [50] and molecular replacement was done with Molrep [51]. A tetramer (biological unit) composed of only main-chain atoms and based on a high resolution X-ray structure of avidin (Airenne, Hytönen *et al. *unpublished; [PDB:1VYO]) was used as a trial model. The best solution (correlation coefficient = 0.291) from molecular replacement was selected as the input for automatic model building with ARP/wARP [52]. After adding side chains separately for each monomer A to H using the guiSIDE mode of ARP/wARP, the model was refined with Refmac5 [53], and modified and rebuilt with O [54]. Solvent atoms were added to the model with the automatic procedure of ARP/wARP [55] and the ligand biotin was built with the ARP/wARP LigandBuild program [56]. Sulfate ions and glycerol molecules were built either manually in O or with the aid of the program Coot [57]. The AVR2-b structure was analyzed with the programs PROCHECK [58] and WHATIF [59]. Structure determination statistics are summarized in Table 3. The coordinates and structure factors of AVR2-b have been deposited in the Protein Data Bank with entry code [PDB:1WBI].

### Biotin dissociation analysis

The dissociation rate constant of AVR2-b, AVR2(I109K), AVR4(K109I), AVR6-b and AVD(K111I)-b for [^3^H]biotin was measured at various temperatures as previously described [60]. [^3^H]Biotin was purchased from Amersham. The data were analysed by using the global fit approach as shown by Hyre *et al. *[41], in which the temperature dependence of the dissociation rate constant is modelled by the Eyring equation.

### Differential scanning calorimetry

The thermal stability of AVR2, AVR2-b, AVR2(I109K), AVR4(K109I), AVR6-b and AVD(K111I) was studied using differential scanning calorimetry (DSC) as previously described [61]. The melting point of protein unfolding was determined from thermograms measured in a buffer containing 50 mM NaPO_4 _(pH 7.0) and 100 mM NaCl. Proteins were also analysed in the presence of biotin (three-fold molar excess of biotin per protein subunit).

### Size exclusion chromatography

Gel filtration experiments were performed as described in Ref. [23] with a Superdex HR 10/30 column using 50 mM NaCO_3 _(pH 11.0), 150 mM NaCl as the liquid phase. The column was calibrated using IgG (158 kDa), BSA (68 kDa) and ovalbumin (44 kDa) as molecular weight standards.

### Mass spectroscopy

The molecular weight of AVR2-b was measured with a Micromass LCT Electronspray ionization TOF Mass spectrometer essentially as described previously [37]. Samples were dialysed against water and diluted 1:1 with acetonitrile. The final protein concentration was 7 μM and the pH was adjusted using formic acid (0.2 %). Positive ions were detected using the default parameters (source temperature 100°C, desolvation temperature 120°C, RF lens voltage 750 V, extraction cone voltage 6 V, sample cone voltage 50 V, capillary voltage 3800 V) and the sample was injected at a rate of 20 μl/min.

### Phylogeny inference

Avidin-related sequences were aligned using MALIGN [62,63]. One-thousand bootstrap variations [64] of the alignment were generated using SEQBOOT and distance matrices produced using a structure-based scoring matrix [62,63]. Trees were produced using NEIGHBOR, and the consensus tree produced using CONSENSE. SEQBOOT, NEIGHBOR and CONSENSE are programs from the PHYLIP package [65,66].

### Miscellaneous methods

The multiple sequence alignment shown in Figure 1A was created using the program MALIGN [62,63] of Bodil [67] and edited with Corel Draw11. The protein representations in Figure 1, 2, 3 were made with the PyMOL Molecular Graphics System [68] and edited with the programs Gimp and/or Corel Draw11. Cavities were calculated with Surfnet [69] using 1.4 Å and 3.0 Å radii for minimum and maximum gap spheres, respectively. The electron density map shown in Figure 1C and 1D was calculated with programs of the CCP4i suite.

## Authors' contributions

The biochemical studies of AVD and AVRs were mainly carried out by VPH and JAEM in the group of Prof. MSK at the University of Jyväskylä and Tampere. JAEM performed also the mutagenesis analyses. HK had an important role in crystallizing the AVR2, whereas KKH and TKMN carried out the DSC analyses. JH had a major role in the expression and purification of the studied AVD and AVRs. TK performed most of the ^3^H-biotin dissociation analyses and MSJ made the phylogenetic inference analysis. The structural analysis of AVR2 was made mainly by TTA in the Structural Bioinformatics Laboratory led by the group leaders MSJ and TAS.

## Figures and Tables

**Table 1:** The recombinant proteins of this study. The immobilised ligand used for affinity chromatography purification and the elution conditions are shown. Protein eluted using acetic acid was immediately dialyzed against 50 mM NaPO_4 _pH 7.0 + 100 mM NaCl.

Protein	Affinity chromatography ligand	Elution conditions	Expression system	Production yield (mg/l)^a^
AVR2	D-biotin	0.5 M acetic acid	BEVS^b^	0.8
AVR2-b	D-biotin	0.5 M acetic acid	*E. coli*^c^	4.9
AVR2(I109K)	D-biotin	0.5 M acetic acid	BEVS	9.0
AVR4^d^	2-iminobiotin	50 mM Na-Ac + 100 mM NaCl	BEVS	6.1
AVR4-b^d^	2-iminobiotin	50 mM Na-Ac + 100 mM NaCl	*E. coli*	21.9
AVR4(K109I)^d^	2-iminobiotin	50 mM Na-Ac + 100 mM NaCl	BEVS	16.2
AVR4(K109I)-b^d^	2-iminobiotin	50 mM Na-Ac + 100 mM NaCl	*E. coli*	6.7
AVR6-b^e^	D-biotin	2 M acetic acid	*E. coli*	8.3
AVD(K111I)	2-iminobiotin	50 mM Na-Ac + 100 mM NaCl	BEVS	11.5
AVD(K111I)-b	2-iminobiotin	50 mM Na-Ac + 100 mM NaCl	*E. coli*	0.7

^a^After affinity chromatography per one liter of culture medium. The yields are calculated based on only a few protein purifications.

^b^Produced using a baculovirus expression system in insect cells as described previously [40].

^c^The bacterial signal peptide from the protein OmpA was utilised in order to produce the protein in an active form in *E. coli *as described in [37].

^d^AVR4 is identical to AVR5. The protein carries the mutation C122S, which prevents oligomerisation *via *intermolecular disulphide bridges [36].

^e^Carries mutation C58S, which prevents oligomerisation *via *intermolecular disulphide bridges (this study).

**Table 2:** Structural properties of avidin and avidin related proteins. Elution times from FPLC gel filtration and calculated molecular weights of the proteins. Heat-induced unfolding temperatures of proteins from DSC analysis (average ± S.D).

	**Gel filtration**		**DSC**		
	
**Protein**	**Elution time (min)**	**Molecular mass (kDa)**	**T_m _(-biotin) (°C)**	**T_m _(+biotin) (°C)**	**ΔT_m_^a ^(°C)**
	
**AVD^b^**	31.5	46.5	83.5 ± 0.1	117.0 ± 0.7	33.5
**AVD(K111I)**	31.9	42.1	76.5 ± 0.1	116.0 ± 0.1	39.5
**AVR2**	30.1	66.6	91.3	112.5	21.2
**AVR2(I109K)**	30.0	68.2	97.6 ± 0.1	118.7 ± 0.1	21.1
**AVR2-b**	30.9	55.1	93.4 ± 0.4	112.5	19.1
**AVR4^c^**	31.9	42.1	106.4 ± 0.8	125.4 ± 0.8	19.0
**AVR4(K109I)**	31.3	49.8	104.5 ± 0.5	118.6 ± 1.2	14.1
**AVR6-b**	31.1	51.5	87.7	114.0	26.3

^a^ΔT_m _is the change in T_m _upon addition of a three-fold molar excess of D-biotin.

^b^DSC results for avidin are from Ref [61].

^c^DSC results for AVR4 are from Ref [36].

**Table 3:** Data collection and structure determination statistics for AVR2.

Data collection^a^	
Wavelength (Å)	0.934
Beamline	ID14-1 (ESRF)
Detector	ADSC Q4R CCD
Resolution (Å)	25 – 1.40 (1.50 – 1.40)
Unique observations	257876 (47505)
I/sigma	14.3 (3.2)
*R*_sym_^b ^(%)	7.2 (54.6)
Completeness	99.6 (99.0)
Redundancy	6.0 (5.9)

Refinement	

Space group	*P*2_1_2_1_2_1_
Unit cell:	
a, b, c (Å)	97.7, 99.9, 135.2
α, β, γ (°)	90, 90, 90
Monomers (asymmetric unit)	8
Resolution (Å)	25 – 1.40
*R*_work_^c ^(%)	17.4
*R*_free_^c,d ^(%)	20.1
Protein atoms	7720
Heterogen. atoms	234
Solvent atoms	1168
*R.m.s.d:*	
Bond lengths (Å)	0.015
Bond angles (°)	1.7
Ramachandran plot:	
Residues in most favored regions	94.3%
Residues in additional allowed regions	5.7%

^a^The numbers in parenthesis refer to the highest resolution bin.

^b^

^c^

^d^Performed on 5% of the reflections.
